# Chemical Composition and Antioxidant Activity of Six *Allium* Extracts Using Protein-Based Biomimetic Methods

**DOI:** 10.3390/antiox13101182

**Published:** 2024-09-29

**Authors:** Ioana Andreea Barbu, Vlad Alexandru Toma, Augustin Cătălin Moț, Ana-Maria Vlase, Anca Butiuc-Keul, Marcel Pârvu

**Affiliations:** 1 Faculty of Biology and Geology, Babeș-Bolyai University, 1, M. Kogălniceanu Street, 400084 Cluj-Napoca, Romania; ioana.barbu@ubbcluj.ro (I.A.B.); anca.keul@ubbcluj.ro (A.B.-K.); marcel.parvu@ubbcluj.ro (M.P.); 2 Doctoral School of Integrative Biology, Babeș-Bolyai University, 400015 Cluj-Napoca, Romania; 3 Center for Systems Biology, Biodiversity and Bioresources, Babeș-Bolyai University, 400084 Cluj-Napoca, Romania; 4 Institute of Biological Research, Branch of NIRDBS Bucharest, 48 Republicii Str., 400015 Cluj-Napoca, Romania; 5 “Maya and Nicolae Simionescu”, Romanian Society for Cell Biology, 050568 Bucharest, Romania; 6 Faculty of Chemistry and Chemical Engineering, Babes-Bolyai University, 11 Arany Janos Street, 400028 Cluj-Napoca, Romania; augustin.mot@ubbcluj.ro; 7 Department of Pharmaceutical Botany, Faculty of Pharmacy, Iuliu Hațieganu University of Medicine and Pharmacy, 8 Victor Babeș Street, 400012 Cluj-Napoca, Romania; gheldiu.ana@umfcluj.ro

**Keywords:** *Allium*, antioxidant effects, cytochrome c, hemoglobin

## Abstract

Medicinal plants are a valuable reservoir of novel pharmacologically active compounds. ROS and free radicals are primary contributors to oxidative stress, a condition associated with the onset of degenerative diseases such as cancer, coronary heart disease, and vascular disease. In this study, we used different spectrophotometry methods to demonstrate the antioxidant properties of 6 *Allium* extracts: *Allium fistulosum*; *Allium ursinum*; *Allium cepa*: Arieș red cultivar of *A. cepa*, and white variety of *A. cepa*; *Allium sativum*; and *Allium senescens* subsp. *montanum*. HPLC–MS determined the chemical composition of the extracts. Among the tested extracts, the Arieș red cultivar of *A. cepa* stands out as having the best antioxidant activity, probably due to the high content of polyphenols and alliin (12.67 µg/mL and 3565 ng/mL, respectively). The results obtained in this study show that *Allium* extracts have antioxidant activity, but also free radical scavenging capabilities. Also, their interactions with cytochrome c and hemoglobin can be the basis of future studies to create treatments for oxidative stress-related diseases.

## 1. Introduction

Medicinal plants are a valuable reservoir of novel pharmacologically active compounds. For instance, natural products are promising alternatives for managing pathogens linked to various diseases [1]. Garlic (*Allium sativum*) and onion (*Allium cepa*), both widely used culinary spices of global significance, have attracted considerable attention for many years. This is primarily due to their extracts and phytochemical components’ broad spectrum of biological activities [2].

Antioxidants play crucial roles in promoting health and are utilized to mitigate the risk of diseases. They possess the capability to shield the body from oxidative damage, which is linked to various diseases like diabetes, cancer, and neurodegenerative disorders. Additionally, antioxidants are effective in regulating oxidative processes, thereby preventing the deterioration of food quality caused by reactive oxygen species (ROS) and free radical reactions within the body [3,4].

ROS and free radicals are primary contributors to oxidative stress, a condition associated with the onset of degenerative diseases such as cancer, coronary heart disease, and vascular disease. Recognizing the hazards posed by ROS and free radical reactions underscores the importance of natural antioxidants in averting oxidative stress. Numerous studies have highlighted the notable antioxidant activity found in the leaves of *Allium* species [3,4,5].

Antioxidant compounds can be sourced from various *Allium* species including *Allium fistulosum*, *Allium ursinum*, *A. cepa*, and *A. sativum*. These compounds are accessible from different plant components such as bulbs, leaves, roots, flowers, and bark [4,6,7]. Polyphenolic compounds represent a category of biologically active substances including phenolic acids and flavonoids found in plant-based foods. Flavonoids are further divided into six subclasses: flavonols, flavones, isoflavones, flavanones, and anthocyanins. Among these, flavanols represent the largest group and are known for their strong antioxidant properties. These compounds serve various roles, including being recognized as nutrients, phytonutrients, plant secondary metabolites, dietary bioactive components, antioxidants, and protective compounds [8,9,10]. Kaempferol and quercetin are the primary types of flavonols, distinguished by the presence of a hydroxyl (OH) group. Both compounds are found in abundance in *Allium* species [11].

Cytochrome c (cyt c) is a critical protein integral to mitochondrial respiratory chains, situated on the outer side of the inner mitochondrial membrane. It serves as a conduit for electrons, transporting them from respiratory complex III (cytochrome c reductase or cytochrome bc1) to the terminal oxygen reductase (complex IV or cytochrome c oxidase). This process is fundamental for cellular maintenance as it enables ATP synthesis, thereby providing energy to living organisms through the electron–proton transfer mechanism. Dysfunction of cyt c can disrupt the mitochondrial respiratory chain and impact the mitochondrial membrane potential (MMP) [12,13].

Hemoglobin (Hb) consists of a tetramer composed of two similar subunits known as α and β chains. The β chains consist of eight helices labeled A to H, whereas the α chains have only seven. Each unit contains a heme group, with a Fe^2+^ ion positioned at the center of the tetrapyrrole ring, coordinated to a histidine residue known as the proximal His. This histidine residue facilitates the reversible binding of oxygen. This binding initiates a sequence of tertiary structural alterations that destabilize the deoxyHb configuration [14,15]. Hemoglobin undergoes continuous autoxidation, resulting in the production of superoxide, which further converts into hydrogen peroxide (H_2_O_2_), serving as a potential source for subsequent oxidative processes. This autoxidation is particularly pronounced under hypoxic conditions within the microcirculation and for unstable dimers formed at decreased Hb concentrations. Within red blood cells (RBCs), oxidative reactions are suppressed by a robust antioxidant mechanism. However, for extracellular Hb, whether originating from RBC hemolysis or the administration of Hb-based blood substitutes, the available antioxidant system does not entirely counteract the oxidative reactions [16]. The oxidation products of hemoglobin represent a potential trigger for oxidative reactions in the plasma, especially pronounced when lower molecular weight Hb dimers penetrate cells and tissues. Studies have revealed that heme and oxyferryl possess pro-inflammatory properties, heightening the risk of oxidative stress. These oxidative reactions play a significant role in various pathological conditions such as atherosclerosis, kidney dysfunction, sickle cell disease, and malaria. The harmful effects of extracellular Hb are particularly concerning in cases of hemolytic anemia, characterized by increased hemolysis [16,17].

Due to the cytotoxic effects of certain medications, researchers have shifted their focus toward plants that have beneficial properties for human health. Thus, in our study, we aimed to demonstrate the interactions of *Allium* extracts with cyt c and hemoglobin.

Starting from the already existing studies that present *A. sativum* and *A. cepa* as having an important antioxidant effect, the purpose of this study was to test the antioxidant properties of six *Allium* extracts (*A. fistulosum*; *A. ursinum*; *A. cepa*: Arieș red cultivar of *A. cepa*, and the white variety of *A. cepa*; *A. sativum*; and *Allium senescens* subsp. *montanum)* using biomimetic methods associated with HPLC–MS analyses. Moreover, data regarding the Arieș red cultivar of *A. cepa* and *A. senescens* subsp. *montanum* and effects of extracts on cyt c and hemoglobin are reported here for the first time.

## 2. Materials and Methods

### 2.1. Extract Preparation

The bulbs of *A. sativum*, Arieș red cultivar of *A. cepa*, and the white variety of *A. cepa* were gathered from a private garden, and leaves of *A. fistulosum*, *A. ursinum*, and *A. senescens* subsp. *montanum* were gathered from the “Alexandru Borza” Botanical Garden in Cluj-Napoca. For each extract, the samples were collected from mature plants, grown without any special conditions. The extracts were prepared using methods detailed in previous publications [18].

### 2.2. Phytochemical Analysis

The *Allium* extracts were thoroughly characterized using two distinct analytical methods validated according to previous studies [19]. The analysis was conducted using an Agilent Technologies 1100 HPLC Series system equipped with an autosampler, column thermostat, binary gradient pump, degasser, and UV detector, which was coupled with an Agilent Ion Trap 1100 SL mass spectrometer (LC/MSD Ion Trap VL, Agilent Technologies, Santa Clara, CA, USA).

The first analytical method was slightly modified by adding five new compound standards to enhance the compound spectrum detection capability [20]. This modified method facilitated the identification of 23 distinct polyphenols in the extracts, namely, apigenin, caffeic acid, 4-O-caffeoylquinic acid, caftaric acid, chlorogenic acid, p-coumaric acid, ferulic acid, fisetin, gentisic acid, hyperoside, isoquercitrin, kaempferitrin, kaempferol, kaempferol-3-rhamnoside, luteolin, myricetin, patuletin, quercetin, quercitrin, rutoside, sinapic acid, vitexin, and vitexin 2-O-rhamnoside. Chromatographic separation was achieved on a Zorbax SB-C18 reverse-phase analytical column (100 mm × 3.0 mm i.d., 3.5 µm particle size, Agilent Technologies, Santa Clara, CA, USA). The mobile phase consisted of a binary gradient of methanol and 0.1% acetic acid (*v*/*v*) in water. The elution started with 5% methanol, increased linearly to 42% methanol over 35 min, followed by an isocratic elution at 42% methanol for 3 min, and then the column was re-equilibrated with 5% methanol over the next 7 min [20]. The flow rate was maintained at 1 mL/min, the column temperature at 48 °C, and the injection volume was 5 µL. Detection of bioactive compounds was performed in both UV and MS modes; the UV detector settings were at 330 nm for up to 17 min for polyphenolic acids and switched to 370 nm for flavonoids and their aglycones for up to 38 min [21]. The mass spectrometer operated using an electrospray ionization source (ESI) in negative mode, with settings including a capillary voltage of +3000 V, a nebulizer pressure of 60 psi (nitrogen), a nitrogen dry gas flow rate of 12 L/min, and a gas temperature of 360 °C.

The second validated LC–MS method targeted the identification of six additional polyphenols, namely, epicatechin, catechin, syringic acid, gallic acid, protocatechuic acid, and vanillic acid, using the same chromatographic conditions as previously described [22]. The mobile phase gradient was slightly different, starting with 3% methanol and increasing to 8% at 3 min, 20% from 8.5 min to 10 min, followed by a re-equilibration to 3% methanol. Detection was exclusively performed in MS mode, with the same ESI source settings as the first method.

For each bioactive compound, MS spectra were compared with reference spectra from an established library to confirm identity. Quantification was carried out using UV detection by employing calibration curves of corresponding standards to quantify peak areas [20,21,22].

For the analysis of alliin in plant extracts, high-performance liquid chromatography coupled with tandem mass spectrometry (LC–MS/MS) was employed, using the same equipment and the same chromatographic column as mentioned above. Elution conditions were optimized with an isocratic mobile phase of 1 mM ammonium acetate, flow rate of 1 mL/min, and a column temperature of 45 °C. The sample injection volume was 2 µL, and the total run time was minimized to 1.4 min to enhance throughput.

Mass spectrometric detection was performed using an electrospray ionization (ESI) source in positive mode. The primary ion monitored was *m*/*z* 178, representing the protonated molecule of alliin, with a subsequent transition to *m*/*z* 88 for quantification, based on specific fragmentation patterns observed in preliminary studies. The ESI conditions were set with a capillary voltage of 3000 V, a nebulizer pressure of 70 psi using nitrogen, and a dry gas flow rate of 12 L/min at 350 °C to ensure efficient ionization and sensitivity [23].

Data acquisition and analysis were conducted using DataAnalysis (v5.3) and ChemStation (vB01.03) software provided by Agilent Technologies. Results were expressed quantitatively as micrograms or nanograms of bioactive compounds per mL of plant extract. This comprehensive analytical approach ensured the precise characterization of the phytochemical profile of *Allium* extracts, contributing valuable insights into their potential therapeutic applications.

### 2.3. Free Radical Scavenging Activity of Allium Extracts

#### 2.3.1. DPPH (2,2-Diphenyl-1-picrylhydrazyl) Assay

A quantity of 2 mg of DPPH (Sigma-Aldrich, St. Louis, MO, USA) was dissolved in 30 mL of 75% ethyl alcohol. Then, 1:1 dilutions were prepared with absolute ethyl alcohol for each extract. Quantities of 400 µL of DPPH and 100 µL of extract were pipetted into the cuvettes, then incubated for 30 min, and the absorbances were read at 530 nm. Quercitin (Sigma-Aldrich) concentrations (0 to 6 µg/mL) were used for the standard curve, and each experiment was performed in triplicate and the results are expressed as mean ± standard deviation [24].

#### 2.3.2. ABTS [2,2′-Azinobis-(3-ethylbenzothiazoline-6-sulfonic acid)] Method

Quantities of 5 mg of ABTS (Sigma-Aldrich) and 30 mg of ammonium peroxydisulfate were weighed and dissolved in 30 mL of distilled water. Then, 450 µL of the previously prepared solution and 50 µL of the 6 extracts were pipetted into the cuvettes. The cuvettes thus prepared were incubated for two hours, and then the absorbances were read at 735 nm. Trolox (Sigma-Aldrich) reagent concentrations (0 to 15 mg/mL) were used for the standard curve (2 mg of Trolox reagent dissolved in 2 mL of 75% ethyl alcohol), and each experiment was performed in triplicate and the results are expressed as mean ± standard deviation [24].

### 2.4. Peroxide Scavenging Activity of the Allium Extracts

The capacity of the tested extracts to scavenge hydrogen peroxide was assessed following this protocol: A solution of H_2_O_2_ (40 mM) was prepared using a phosphate buffer (Sigma-Aldrich) at pH 7.4. Various concentrations of the extracts (ranging from 0.5 to 50 μL/mL of each extract) and ferulic acid (used as control) were mixed with 3.4 mL of phosphate buffer and 0.6 mL of H_2_O_2_. The absorbance of the resulting reaction mixture was measured at 230 nm. A blank solution containing only the phosphate buffer and the extracts was also prepared. The percentage of H_2_O_2_ scavenged by the tested extracts was calculated using the following formula: % of scavenged H_2_O_2_ = [(A0 − A1)/A0] × 100, where A0 represents the absorbance of the control (phosphate buffer with H_2_O_2_) and A1 represents the absorbance of the tested extracts [25]. Each experiment was performed in triplicate and the results are expressed as mean ± standard deviation.

### 2.5. Interaction of Allium Extracts with Cytochrome c and Hemoglobin

From the stock solution of cyt c (1000 μg/mL), the working solution with a concentration of 20 μg/mL was prepared in a phosphate buffer. Then, 1 mL of the solution obtained was mixed with each extract at concentrations of 10 μL/mL, 20 μL/mL, and 50 μL/mL. Also, 1 mL of cyt c was mixed with ascorbic or ferulic acid at the three concentrations. The samples were incubated for one hour at room temperature and then were read on a spectrophotometer at wavelengths between 200 and 700 nm [13]. The same protocol was repeated for the interaction with hemoglobin. Extract interaction with cyt c and hemoglobin was also tested one hour after adding H_2_O_2_ (3% concentration) to the mixture. Every trial was conducted three times, and the outcomes are presented as the mean value ± the standard deviation.

### 2.6. Hydroxyl Radical Scavenging of Allium Extracts

The hydroxyl radical scavenging activity of the extracts was determined as follows: *Allium* extracts were mixed with iron–EDTA solution (0.13% ferrous ammonium sulfate + 0.26% EDTA), EDTA solution, DMSO (dimethyl sulfoxid 0.85% in 0.1 mol/L phosphate buffer pH 7.4), and ascorbic acid. The concentrations of *Allium* extracts used were 10 μL/mL, 20 μL/mL, and 50 μL/mL. The mixture was then heated in a water bath at 80–90 °C for 15 min and subsequently cooled by adding ice-cold TCA (Trichloroacetic acid 17.5%). Nash reagent (7.5 g of ammonium acetate, 0.5 mL of glacial acetic acid, 0.2 mL of acetylacetone, and distilled water to a total volume of 100 mL) was added to the reaction mixture, followed by incubation at room temperature for 15 min for color development. The intensity of the yellow color formed was measured at 412 nm against a reagent blank. Standards such as ascorbic acid and ferulic acid were employed. All substances were purchased from Sigma-Aldrich. The percentage of inhibition was calculated using the following formula: %HRSA = ((Abs__control_ − Abs__sample_) × 100)/Abs__control_ [26]. Every trial was conducted three times, and the outcomes are presented as the mean value ± the standard deviation.

### 2.7. Interaction with Bovine Serum Albumin (BSA) of Allium Extracts

A solution of 0.001 mM albumin in 0.1 M Tris-HCl, pH 7.4, was prepared. Two milliliters of the BSA solution were aliquoted into spectrophotometer cuvettes, onto which 10 μL, 20 μL, and 50 μL of each of the 6 extracts were added. They were then incubated for 15 min and subsequently read on the spectrophotometer at a wavelength of 277 nm.

### 2.8. Statistical Analysis

For statistical analyses, the GraphPad Prism 8 software was used, and to highlight significant differences between groups, the two-way ANOVA test was applied.

## 3. Results

### 3.1. Free Radical Scavenging Activity of Extracts

The ABTS and DPPH assays are commonly employed techniques for evaluating the antioxidant capacities of natural products. They both utilize spectrophotometric methods centered on the attenuation of stable colored radicals (ABTS or DPPH). These assays demonstrate the ability of antioxidants to scavenge radicals, even in intricate biological mixtures like plant or food extracts. As shown in Table 1, the highest antioxidant activity is exhibited by the extract of Arieș red cultivar of *A. cepa*, as demonstrated by both the DPPH and ABTS methods. The extract of *A. ursinum*, and that of the white variety of *A. cepa*, displayed lower antioxidant activity. The extracts demonstrated similar results across both methods, but the antioxidant effect was greater when the ABTS protocol was used compared to DPPH.

### 3.2. Hydroxyl Radical Scavenging

**Figure 1:**
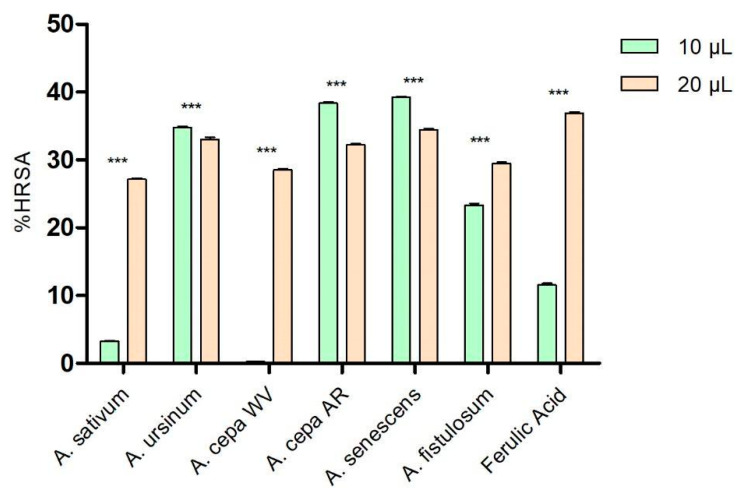
Hydroxyl radical scavenging activity (%HRSA) by *Allium* extracts at two different volumes: 10 μL and 20 μL. 1. *A. sativum*, 2. *A. ursinum*, 3. white variety of *A. cepa*, 4. Arieș red cultivar of *A. cepa*, 5. *A. senescens* subsp. *montanum*, 6. *A. fistulosum*, and 7. Ferulic acid; *** *p* < 0.001, according to the two-way ANOVA and Bonferroni’s post-test.

As shown in Figure 1, at low concentrations (10 μL), the extracts of *A. ursinum*, Arieș red cultivar of *A. cepa*, *A. senescens*, and *A. fistulosum* showed higher percentages of hydroxyl radical scavenging (34.77 ± 0.14%, 38.36 ± 0.35%, 39.21 ± 0.24%, and 23.32 ± 0.49%, respectively) compared to ferulic acid (11.56 ± 0.45%), used as a control because of its antioxidant effects. The white variety of *A. cepa* (0.26 ± 0.06%) and *A. sativum* (3.29 ± 0.14%) demonstrated significantly lower antioxidant activity than the control. At a higher concentration (20 μL), the extract of *A. senescens* (34.43 ± 0.33%) was the closest to the control (36.91 ± 0.24%), followed by *A. ursinum* and Arieș red cultivar of *A. cepa*, while *A. sativum* remained the extract with the lowest hydroxyl radical scavenging capacity among the analyzed extracts. Based on the results of the two-way ANOVA statistical analysis, all values demonstrate statistical significance with a *p*-value of less than 0.001 when compared to the control group.

### 3.3. Peroxide Scavenging Activity

**Figure 2:**
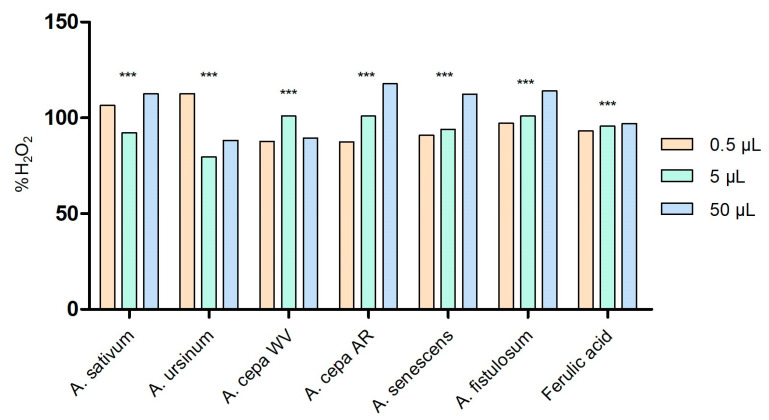
Peroxide scavenging activity of *Allium* extracts at three different concentrations: 0.5 μL, 5 μL, and 50 μL. 1. *A. sativum*, 2. *A. ursinum*, 3. white variety of *A. cepa*, 4. Arieș red cultivar of *A. cepa*, 5. *A. senescens* subsp. *montanum*, and 6. *A. fistulosum*; ferulic acid was used as standard peroxide scavenger molecule; *** *p* < 0.001, according to two-way ANOVA and Bonferroni’s post-test.

The capacity of the *Allium* extracts to scavenge hydrogen peroxide is illustrated in Figure 2. All extracts exhibited the ability to neutralize H_2_O_2_ in a dose-dependent manner. At low volume (0.5 μL), the extract that stood out for its highest capacity to scavenge H_2_O_2_ was *A. ursinum* (112.626 ± 0.05%). However, as the concentration increased, this capacity decreased. By contrast, the Arieș red cultivar of *A. cepa* extract showed better effects at high concentrations (50 μL; 117.857 ± 0.0002%), but the effect decreased as the concentration decreased. The same pattern was observed for the extracts of *A. senescens* and *A. fistulosum*. When the scavenging % is more than 100, the radical scavenging is more than 1 unit and the extract is a potent antioxidant. In our case, a combination of antioxidants may have an additive effect, leading to enhanced radical scavenging. This might not technically “exceed” 100%, but due to an additive or potentiating effect, the system may appear to scavenge more than the calculated amount of radicals based on individual components. According to the two-way ANOVA and Bonferroni’s post-test, all values are statistically significant with *p* < 0.001.

### 3.4. Interaction with Hemoglobin and Cytochrome c of Allium Extracts

**Figure 3:**
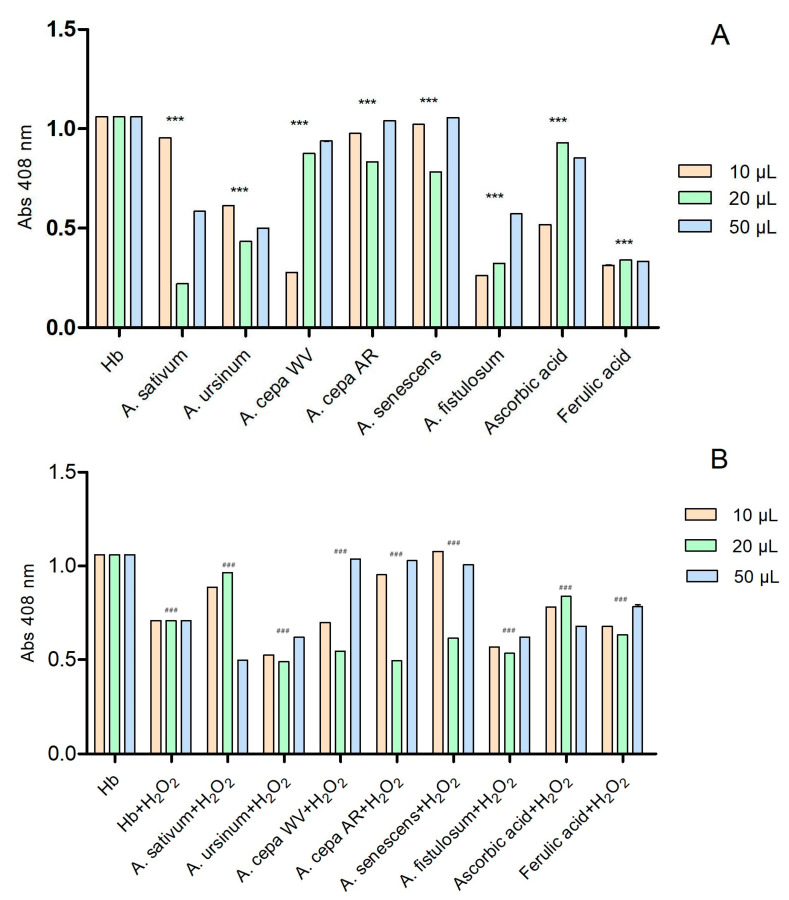
The interactions of the extracts with hemoglobin (**A**) and with hemoglobin after adding 6 μL of H_2_O_2_ (**B**); in the presence of hydrogen peroxide, hemoglobin may exhibit a catalase-like function, breaking down H_2_O_2_ into water and oxygen, which prevents further oxidative damage. These reactions apparently suggested that hydrogen peroxide plays an antioxidant function; ascorbic and ferulic acid were used as antioxidant standards. *** *p* < 0.001 (**A**), ### *p* < 0.001 (**B**) according to two-way ANOVA and Bonferroni’s post-test.

The six extracts interact with hemoglobin in a concentration-dependent manner (Figure 3A). Ascorbic acid and ferulic acid were used as controls due to their known antioxidant effects. We also considered their antioxidant effect to be the maximum. The extracts that had absorbance values higher than the two standards showed a prooxidative character. At the lowest tested concentration (10 μL), the extracts of the white variety of *A. cepa* (*A. cepa* AR) and *A. fistulosum* exhibited hem-mediated antioxidant activity similar to ferulic acid, while *A. ursinum* showed activity similar to ascorbic acid. On the contrary, the extracts of *A. sativum*, Arieș red cultivar of *A. cepa*, and *A. senescens* subsp. *montanum* demonstrated prooxidative action. At a concentration of 20 μL, all six extracts showed antioxidant activity. The extracts of the white variety of *A. cepa* (*A. cepa* WV), Arieș red cultivar of *A. cepa*, and *A. senescens* subsp. *montanum* exhibited antioxidant properties similar to ascorbic acid, but at the highest concentration (50 μL), they showed prooxidative activity. In Figure 3B, the effect of the extracts on hemoglobin after adding 6 μL of H_2_O_2_ was observed. Ascorbic acid and ferulic acid were also used as controls, and the extracts had similar effects except for *A*. *sativum*, which only exhibited antioxidant effects at 50 μL. At 20 μL, the extracts that had prooxidant effects showed the opposite effect in the presence of hydrogen peroxide.

**Figure 4:**
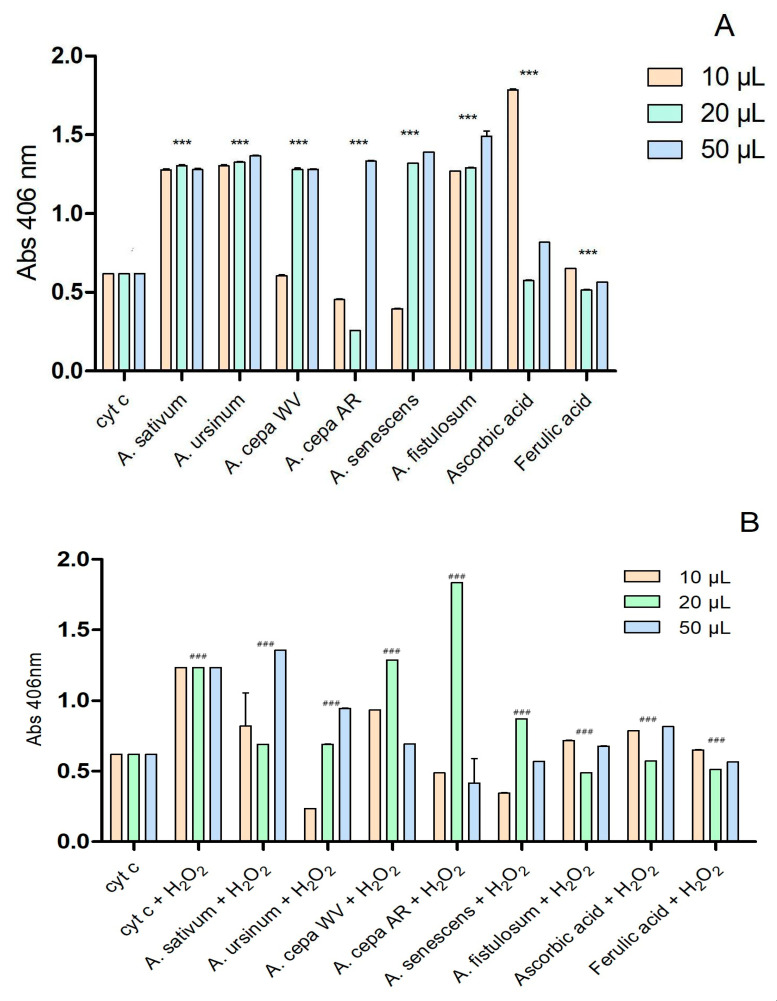
Extracts’ interactions with cytochrome c (**A**) and cytochrome c after adding 2 μL of H_2_O_2_ (**B**); ascorbic and ferulic acid were used as antioxidant standards; *** *p* < 0.001 (**A**), ### *p* < 0.001 (**B**), according to two-way ANOVA.

The interaction of the extracts with cytochrome c is presented in Figure 4. The two extracts of *A. cepa* and the one of *A. senescens* subsp. *montanum* exhibited antioxidant activity similar to that of ferulic acid at a concentration of 10 μL. At higher concentrations (20 μL and 50 μL), all extracts except for the Arieș red cultivar of *A. cepa* (20 μL) showed prooxidant effects.

After adding 2 μL of H_2_O_2_ (Figure 4B), the Arieș red cultivar of *A. cepa* exhibited a highly pronounced prooxidative character at 20 μL, compared to ascorbic acid and ferulic acid. Depending on the concentration, the extracts of the white variety of *A. cepa* and *A*. *sativum* showed prooxidative effects. The other extracts had effects similar to the controls used. According to the statistical analysis using two-way ANOVA, all values show statistical significance with a *p*-value below 0.001 when compared to the control group.

### 3.5. Measurement of the Affinity of Allium Extracts for Plasma Proteins

The binding constants of Allium extracts with bovine serum albumin refer to the strength of the interaction between these molecules. They quantify how tightly or weakly *Allium* extracts bind to bovine serum albumin. From Table 2, it can be observed that the six extracts form relatively stable complexes with albumin (k~1) compared to ascorbic acid and chloramphenicol. Values close to 1 indicate only one association zone between the extract and the protein. The strongest bond is exhibited by the extract of Arieș red cultivar of A. *cepa* (1.6123), owing to the high concentration of polyphenols in the extract. The weakest interaction with albumin is observed in the extract of the white variety of *A. cepa* (0.8954).

### 3.6. Phytochemical Analysis of the Extracts

Table 3 presents the chemical composition of polyphenols and alliin in the six *Allium* extracts. The highest quantity of alliin is found in the extract of *A. sativum* (11,680.28 µg/mL), followed by the Arieș red cultivar of *A. cepa* (3565.17 µg/mL). *A. ursinum* and *A. senescens* subsp. *montanum* have the lowest concentrations of alliin. Gentisic acid, rutin, luteolin, and kaempferol were not detected in any of the extracts using the method employed. Para-coumaric acid is present in the sample of *A. ursinum*, while isoquercetin is found in the extracts of the white variety of *A. cepa*, Arieș red cultivar of *A. cepa*, and *A. senescens* subsp. *montanum*, and quercetin is found in the extracts of *A. ursinum*, and *A. fistulosum*.

## 4. Discussion

Studies have shown that ROS can cause various diseases such as heart disease, cancer, or pathologies associated with aging, such as Parkinson’s and Alzheimer’s. *Allium* species have proven to have an important role in maintaining the health of the human population due to compounds that have different effects, such as antimicrobial [18,27], anti-inflammatory [28], or antioxidant effects [4].

The antioxidant effects of the six extracts are closely related to their chemical composition. Previous studies about *Allium* extracts showed that red onion has a higher content of polyphenols than garlic (86 ± 1.00 mg GAE/100 g compared to 45 ± 1.00 mg GAE/100 g); likewise, alliin content is higher in *Allium sativum* extracts compared to other extracts, a fact also noticed in our studies [18,29]. Stupar (2021) analyzed the chemical composition of *A. ursinum* and obtained concentrations of kaempferol derivatives ranging from 8.96 µg/mL to 29.95 µg/mL [30]. In our study, the extract of *A. ursinum* had a concentration of kaempferol below the detection limit of the method. The chemical composition of the extracts also varies depending on the species, the period of plant harvesting, but also on the solvent used to obtain the extract, as was noticed in previous studies [30,31,32].

DPPH and ABTS radicals are considered stable free radicals and can be effectively reduced by glutathione, ascorbate or sulfur-containing amino acids like cysteine. Consequently, these radicals are extensively utilized for evaluating the in vitro antioxidant capacity of different extracts. Bozin et al. (2008) noticed that this capacity of garlic extract depends on the age of the plant. Extracts from immature plants had the best antioxidant effects (84.7% of neutralization of the DPPH radical by garlic extracts) compared to those obtained from mature plants (44.8% from fresh bulbs and 57.6% from dried bulbs) [20]. Ye et al. (2013) also noticed the antioxidant effects of *Allium cepa* oil [33]. Lee et al. (2021) analyzed the antioxidant effects of four aqueous extracts of *Allium* (*A. tuberosum*, *A. senescens*, *A. thunbergii*, and *A. sacculiferum*), and their results for the *A. senescens* extract were comparable to our data, with the extract showing a high capacity to reduce the DPPH radical. Additionally, the *A. senescens* extract stood out in the ABTS method as one of the extracts with the highest capacity to reduce the ABTS radical [34]. Fidrianny (2013) tested three different onion extracts (two ethyl acetate extracts, these being red onion and white onion, and one hexane extract). He noticed that both ethyl acetate extracts had better antioxidant effects than the hexane extract using both methods. Additionally, the ethyl acetate extract with red onion showed stronger antioxidant activity than that of white onion [35]. Our study showed the stronger antioxidant activity of red onion, which could be explained by the high concentration of polyphenols [18].

Hydroxyl radical (HO^−^) is an extremely reactive free radical generated in biological systems and has been identified as a highly destructive species in free radicals-associated pathologies. Conversely, H_2_O_2_, while not a free radical itself, is a biologically significant oxidant due to its capacity to produce HO^−^. Moreover, owing to its non-ionized and low charge state, H_2_O_2_ can easily cross cell membranes and instigate toxic effects. Consequently, it is advantageous for cells to regulate and inhibit the buildup of H_2_O_2_. The extract of *A. hookeri* had concentration-dependent HO^−^ scavenging capacity, comparable to ascorbic acid (35–80% and 50–90%, respectively) with an increase in the inhibition percentage as the concentration increases [36]. In their study, Zhang (2015) showed that fresh garlic exhibited a low peroxide scavenging activity of 3.1 ± 0.03%. However, the scavenging capacity of black garlic consistently increased to 30.8% with an increase in heating time from 0 to 10 days. This finding indicates that the peroxide scavenging activity of the black garlic extracts was tenfold higher compared to that of the fresh garlic extracts [37]. In our study, we observed an increase in the ability to scavenge hydroxyl radicals with an increase in the extract concentration. In this study, the extracts showed hydroxyl radical scavenging percentages between 0.26 and 39 at low volumes, and between 27.15 and 34.43 at higher volumes, varying depending on the type of extract. Higher volumes (here, the volumes were assumed as concentrations) of the extract had activities comparable to ferulic acid, used as a control. Other studies showed that different solvents were used to obtain the extracts, but also that the concentration influenced hydrogen peroxide scavenging. The aqueous extracts had higher inhibition percentages than the petroleum ones (67–73% compared to 23–35%) [38]. These results are comparable to those presented in this study, where higher concentrations of the extract had higher percentages of hydrogen peroxide scavenging (88.33–117.85%).

Ascorbic acid has a dual effect. It can convert ferric cytochrome c (cyt c) to its ferrous form [39]. Additionally, hydrogen peroxide degrades cyt c, releasing heme iron and thereby preventing ascorbic acid from reducing ferric cyt c. In turn, ascorbic acid is capable of reducing Fe^3+^ to Fe^2+^. Ferrous ions, when combined with hydrogen peroxide, form potent oxidants such as hydroxyl radicals and/or ferryl components. The Fe^2+^/H_2_O_2_ system has been demonstrated to induce cardiolipin fragmentation [40]. The involvement of free radicals in cyt c/H_2_O_2_ system-mediated DNA cleavage was confirmed by the observation that radical scavengers like ethanol and mannitol significantly inhibit this process [41]. Ferulic acid has the property of stabilizing the cyt c molecule and thus preventing apoptosis induced by cyt c in the case of the hepatoma cell line SMMC-7721 [42]. Our experiments showed that the six tested extracts have concentration- and species-dependent effects, but are comparable to those of ferulic and ascorbic acid used as controls. Also, in the presence of hydrogen peroxide, the extracts had cyt c protection effects, but also prooxidant effects at high concentrations (white variety of *A. cepa* and *A. sativum* at 20 µL and 50 µL, respectively).

Most protective flavonoids demonstrate effectiveness in the 1–10 μM range in vitro, particularly in cell death models where mitochondrial dysfunction and intrinsic apoptosis are involved or evident, such as through caspase-9/-3 activation, mitochondrial depolarization, or the release of cyt c from mitochondria. This consistent concentration range, regardless of the flavonoid class, does not correspond to their varying abilities to scavenge free radicals [43,44]. Epicatechin, kaempferol, and quercetin were capable of reducing cyt c. These three flavonoids produced similar characteristic changes in the absorption spectra of cyt c, as observed with known reductants like dithionite (sodium hydrosulfite) or ascorbate [43,45]. Flavonoids have effects on cyt c depending on the concentration, with higher concentrations producing stronger effects [43]. These results are similar to those obtained in our study due to the high flavonoid content in the extracts. Extracts with a high flavonoid content (Arieș red cultivar of *A. cepa* and *A. senescens* subsp. *montanum*), at low concentrations, showed an antioxidant effect comparable to that of ascorbic acid used as a control. Ascorbic and ferulic acids are recognized as stable and readily available reagents that have demonstrated their effectiveness in eliminating reactive oxygen species (ROS). The same effect was observed in the interaction with hemoglobin at 408 nm [42,46]. Henneberg (2013) demonstrated that quercetin, rutin, hesperidin, and myricetin exhibited antioxidant effects against the production of reactive oxygen species depending on the concentration. The production of reactive oxygen species was significantly inhibited when red blood cells were pre-incubated with these flavonoids, in both healthy individuals and patients with sickle cell anemia. Quercetin and rutin showed the highest antioxidant activity even at low concentrations (30 μmol/L), followed by myricetin and hesperidin, which showed antioxidant properties at a concentration of 100 μmol/L [47,48]. In our study, we demonstrated the effects that *Allium* extracts have on hemoglobin compared to those of vitamin C and ferulic acid. The results showed that depending on the concentration, the extracts can have a protective effect on hemoglobin by protecting its oxidation into methemoglobin (the white variety of *A. cepa* and *A. fistulosum* extracts), or a prooxidant action, such as in *A. sativum*, Arieș red cultivar of *A. cepa*, and *A. senescens* subsp. *montanum*. The prooxidant effect of the extracts, demonstrated at high concentrations, may be due to the presence of ethanol, which was used as a solvent in the extraction process or, according to other studies, due to high amounts of antioxidants displaying prooxidant behavior based on Fenton reactions, inappropriate redox cycling and/or redox system overload [49,50]. Also, as in many studies with plant extracts, the presence of alcohols is one of the limitations of this study. Previously, researchers tested different polyphenols, especially flavonoids, to demonstrate their antioxidant effects. Starting from this research and knowing the high polyphenol content of *Allium* extracts, in this study, we showed for the first time their antioxidant potential on cyt c and hemoglobin.

To estimate the in vivo activity and pharmacokinetic profiles of the extracts, measurement of the albumin-binding capacity was designed as a confident output. For instance, human serum albumin (HSA) plays a role in binding and transporting various drugs, hormones, and compounds like fatty acids in the bloodstream [51]. Due to its high structural similarity to human serum albumin (HSA), bovine serum albumin (BSA) has been extensively studied as a model protein in various fields [52]. To pave the way for new treatments and applications involving plant extracts, we also calculated the affinity constant of the six extracts with bovine serum albumin, comparing these values with vitamin C and chloramphenicol as internal references, observing that they form a single zone of association with albumin while the affinity constant has values close to 1. The findings were the following: the extract of Arieș red cultivar of *A. cepa* has the highest binding constant, while the extract of the white variety of *A. cepa* has lowest. These data were also reported for different extracts of *Azadirachta indica* [53] or different flavonoids such as quercetin (4.94 × 10^5^) or rutin (1.65 × 10^4^) [54]. Taken together, the data show bioactivities of *Allium* species that have not been highlighted by previous research. Testing them using biomimetic chemical systems suggests the possible in vivo behavior of the extracts. Regarding their redox-modulating action, the phenomenon was found to be dose-dependent but also influenced by the specific *Allium* species.

## 5. Conclusions

The results obtained in this study show that *Allium* extracts have antioxidant potential, but also free radical scavenging capabilities. Also, it shows for the first time the effects that *Allium* hydroalcoholic extracts have on cyt c and hemoglobin. These interactions may form the basis of future studies to create treatments for diseases involving oxidative stress. The antioxidant effect of the hydroalcoholic extracts obtained from different species of *Allium* is dependent on the concentration and the plant from which it was obtained. It can be associated with the presence of bioactive compounds, namely, polyphenols and alliin; thus, the extract of Arieș red cultivar of *A. cepa* stood out among the six tested extracts.

## Figures and Tables

**Table 1:** In vitro, antioxidant activity was determined using DPPH and ABTS scavenging assays.

Extracts	ABTS (μg TE/g)	DPPH (μg QE/g)
*A. sativum*	43.4 ± 8.0	15.1 ± 1.3
*A. senescens* subsp. *montanum*	81.8 ± 5.8	40.0 ± 2.3
*A. fistulosum*	34.8 ± 2.0	20.7 ± 0.5
Arieș red cultivar of *A. cepa*	124.7 ± 4.2	49.7 ± 1.6
white variety of *A. cepa*	40.6 ± 1.6	11.3 ± 1.0
*A. ursinum*	36.9 ± 0.3	11.0 ± 1.2

ABTS—TE—trolox equivalents; DPPH—QE—quercetin equivalents.

**Table 2:** Affinity constant of *Allium* extracts with albumin.

*A. sativum*	*A. ursinum*	White Variety of *A. cepa*	Arieș Red Cultivar of *A. cepa*	*A. senescens* subsp. *montanum*	*A. fistulosum*	Ascorbic Acid	Chloramphenicol
1.0508	1.0577	0.8954	1.6123	1.0146	0.9608	0.3315	0.6585

**Table 3:** Phytochemical analysis (HPLC) of the *Allium* extracts. The values are indicated as µg analyte per mL extract.

Class	Compounds	*A. sativum*	*A. ursinum*	White Variety of *A. cepa*	Arieș Red Cultivar of *A. cepa*	*A. senescens* subsp. *montanum*	*A. fistulosum*
Polyphenols (µg/mL)	Gentisic acid	-	-	-	<LOQ	-	-
	p-cumaric acid	-	1.965 ± 0.176	-	-	-	-
	Isoquercetin	-	-	1.317 ± 0.158	3.475 ± 0.104	1.317 ± 0.065	-
	Rutozid	-	-	-	-	-	<LOQ
	Quercetin	-	3.347 ± 0.133	<LOQ	-	<LOQ	8.582 ± 1.115
	Luteolin	<LOQ	<LOQ	<LOQ	<LOQ	<LOQ	<LOQ
	Kaempferol	<LOQ	<LOQ	<LOQ	<LOQ	<LOQ	<LOQ
	Protocatechuic acid	38.065 ± 5.329	4.774 ± 0.238	0.168 ± 0.023	9.211 ± 0.460	1.481 ± 0.088	-
Alliin (ng/mL)		11,680.282 ± 487.213	88.601 ± 6.128	796.048 ± 92.792	3565.178 ± 187.975	94.769 ± 12.856	200.345 ± 18.573

<LOQ—below the quantification limit of the analytical method.

## Data Availability

Data are contained within the article.
